# Assessing the Impact of Celiac Disease on the Quality of Life in Jordan

**DOI:** 10.7759/cureus.74395

**Published:** 2024-11-25

**Authors:** Laith M Haj-Ahmad, Abida Alqaisi, Eyad Altamimi

**Affiliations:** 1 College of Medicine, The University of Jordan, Amman, JOR; 2 Language and Translation Department, The Celiac Care Provider Society, Amman, JOR; 3 Pediatric Gastroenterology Department, King Abdullah University Hospital, Ar-Ramtha, JOR

**Keywords:** adolescents, celiac disease, gluten-free diet, growth problems, jordan

## Abstract

Introduction: Celiac disease profoundly impacts individuals' daily lives, prompting the need to assess health-related quality of life (HRQoL) in affected children. This study aimed to evaluate HRQoL among Jordanian children with celiac disease, considering sex, dietary adherence, presence or absence of growth issues, concurrent chronic diseases, and duration since diagnosis.

Methods: A cross-sectional study invited children from the Celiac Disease Care Providers Society to complete an online Kidscreen-52 questionnaire in Arabic. Descriptive statistics and T-scores for 10 health domains were analyzed against international benchmarks. Cohorts were stratified by concurrent diseases, disease duration, dietary adherence, and growth issues, with comparisons made using t-tests and Cohen's ds.

Results: Among 400 registered members, 126 children (31.5%) participated. Males exhibited inferior HRQoL in multiple domains compared to the general population, with recent studies corroborating findings in females. Males with chronic diseases reported significantly worse moods and self-perception. Females with growth issues faced academic challenges and financial constraints, while those not strictly adherent to a gluten-free diet reported strained parental relationships and challenges in their home environment.

Conclusion: This study reveals a marked decline in HRQoL among Jordanian children with celiac disease, emphasizing the need for educational initiatives targeting patients and healthcare providers, alongside broader advocacy for gluten-free diet support. Further research is imperative to identify additional contributing factors and implement community-level interventions.

## Introduction

Celiac disease is a chronic immune-mediated enteropathy that tends to occur in genetically susceptible individuals upon exposure to dietary gluten. A myriad of gastrointestinal and extraintestinal complications are associated with celiac disease, with recent trends in pediatric patients favoring the latter [1]. Extraintestinal symptoms can happen in any organ, which shows how important it is to be very suspicious of celiac disease when dealing with symptoms that do not seem to be related to the intestines [2].

Among those who have mucosal lesions, a strict gluten-free diet (GFD) is the only effective treatment available. However, the GFD does come with its own realm of misfortune, ranging from a lack of compliance to nutritional complications. A wide spectrum of factors can contribute to dietary non-adherence. The need to adhere to a set of predetermined foods causes social anxiety in this age group of self-conscious teenagers, which is a major concern. Furthermore, the lack of immediate health effects from small amounts of gluten can lead some children to abandon this regimen [3]. In addition, a GFD predisposes patients to a set of nutritional complications. This ranges from the overconsumption of macronutrients to micronutrient deficiencies. Gluten, a protein with minimal caloric value, plays a vital role in the texture and structure of many processed foods. Its removal often results in increased carbohydrate and fat content in gluten-free alternatives. Without proper guidance from an experienced nutritionist, this can lead to obesity or overweight, which are known to negatively affect health-related quality of life (HRQoL). Moreover, many gluten-free products lack age-appropriate vitamins and minerals that are naturally present in otherwise gluten-rich foods, making nutritional deficiency another concern. Overall, even though GFD is the only therapeutic regimen whose effectiveness has received overwhelming consensus, it still imposes its own issues, underscoring the significance of a study of the quality of life of children with celiac disease [4].

Why is this important? HRQoL is a synthesis of one's perceptions of their physical, mental, and social well-being, as well as how they function daily with respect to these domains [5]. Given that celiac disease is a chronic entity with numerous implications for all the previously mentioned domains, a proper assessment of quality of life is crucial.

For several years, researchers have assessed the HRQoL of pediatric patients with celiac disease. Generally, we classify the questionnaires used in this regard into generic and disease-specific categories. In our study, we used the cross-cultural Kidscreen-52 generic validated questionnaire, which was synthesized by 13 European countries. Two versions of this self-report questionnaire are available: one for the children to self-assess, and another for the patient's parents to complete as a proxy. This long version of the Kidscreen questionnaire involves 10 domains, namely, physical well-being, psychological well-being, moods and emotions, self-perception, autonomy, parent relations and home life, financial resources, peers and social support, school environment, and bullying [6].

Nikniaz and colleagues conducted a 2020 meta-analysis, which was the first to systematically review studies that assessed quality of life among children with celiac disease [7]. Only one paper by Myléus et al., out of the 26 included studies, utilized the Kidscreen-52 questionnaire for their assessment. This meta-analysis's lack of inclusion of studies from the Arab world underscores the necessity of this initiative [8].

This is, to the best of our knowledge, the first study to address the quality of life among Jordanian children diagnosed with celiac disease. As demonstrated by two previous studies in our region, celiac disease is not uncommon in Jordan. However, the true prevalence of celiac disease appears to be underappreciated due to physicians' lack of knowledge regarding its wide spectrum of clinical presentations [9,10]. The high prevalence of celiac disease in our region and the need for evidence-based data to inform legislation in Jordan that promotes GFD awareness highlight the necessity of this study. This study aimed to evaluate HRQoL among Jordanian children with celiac disease, considering sex, dietary adherence, presence or absence of growth issues, concurrent chronic diseases, and duration since diagnosis.

## Materials and methods

Questionnaire

Designed to evaluate patients' quality of life, both healthy and ill, between the ages of 8 and 18, the Kidscreen-52 is a validated generic cross-cultural questionnaire. With the intention of being a cross-cultural tool, it was developed in 13 different European nations. We used the Arabic version, which is one of the 38 languages available for the questionnaire [11]. Previous research has proven the acceptable validity, reliability, and internal consistency of the questionnaire's items. Three versions of the Kidscreen instrument exist, with the Kidscreen-52 serving as the most comprehensive version in this study. It covers 52 questions across 10 dimensions ranging from physical health to bullying, as mentioned previously. The questionnaire presents each item on a 5-point Likert scale, weighing the lowest response at 1 and the highest response at 5. The questionnaire's 52 items measure parameters based on the frequency of behaviors or feelings or, less frequently, attitude intensity. The higher the HRQoL, the higher the score [12].

We then add up the item scores from each subscale to create a Rash person parameter. This parameter proves invaluable since it uses an interval scale rather than an ordinal Likert scale. This finding suggests a deeper understanding and greater significance of the actual difference between the two scores. This parameter is z-standardized and converted to its corresponding T value. Next, we need to compare the T value with the normative data of the relevant population. For each of the 13 European countries, normative data are available both separately and collectively [6]. Unfortunately, there is no such data available for the Jordanian population, so we compared cohorts within our study sample to one another and used European values as a proxy.

Study participants

**Figure 1:**
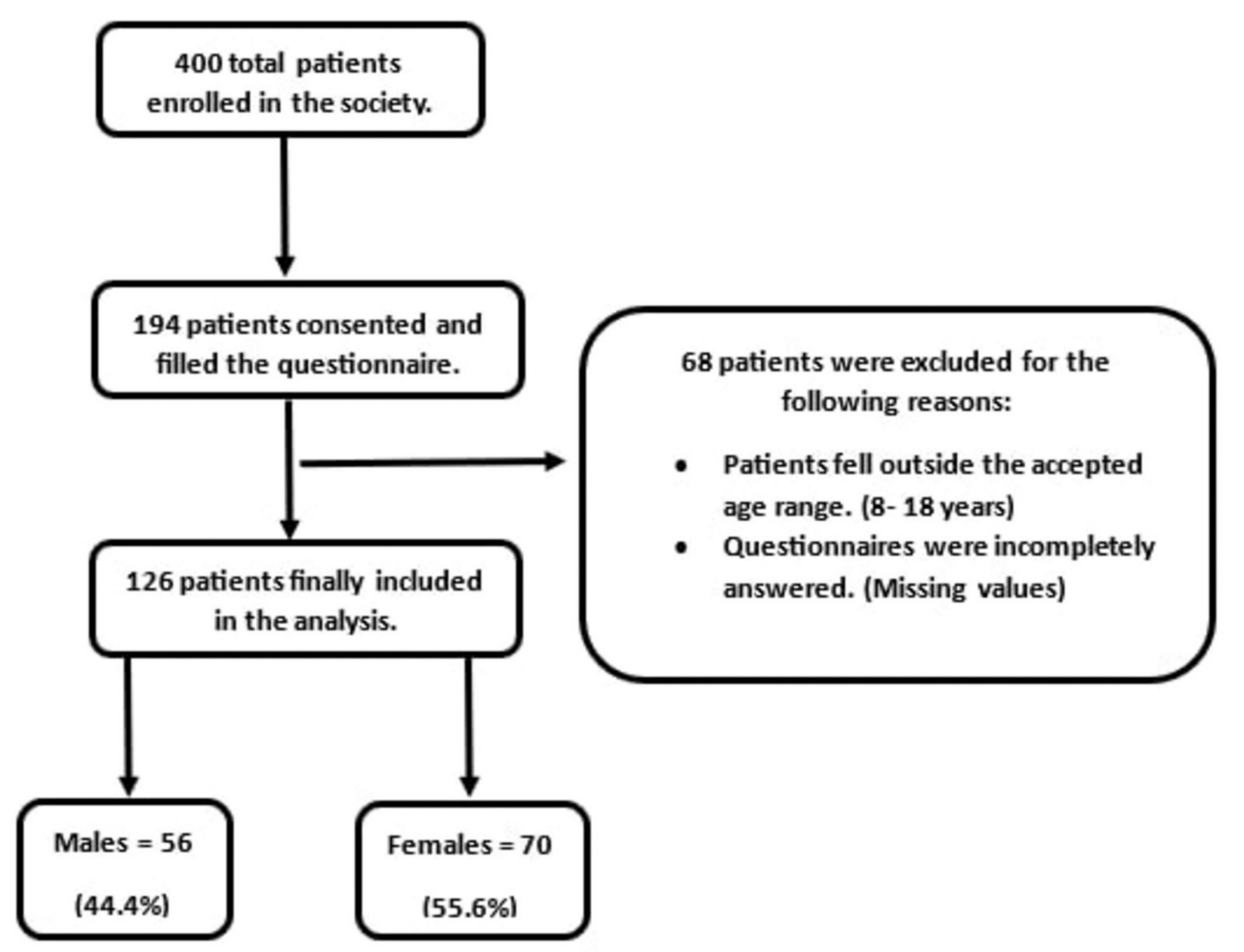
Study participants Out of the 400 patients enrolled in the Celiac Care Provider Society (CCPS), 194 consented and filled in the questionnaire. Sixty-eight patients were excluded due to reasons clarified in the figure. The final number of patients available for analysis was 126 patients, 56 of whom were males and 70 of whom were females.

The recruitment of participants was made with the help of the Celiac Care Provider Society (CCPS), which maintains a registry of patients diagnosed with celiac disease in Jordan. A total of 400 patients were approached to independently complete the self-assessment version of the Kidscreen-52 questionnaire using convenience sampling. Over a few months, the organization presented the questionnaire and frequently reminded the patients to complete it. The data was collected from 21 February 2023 to 12 May 2023. We informed the patients that completing the questionnaire would take them approximately 15-20 minutes, and we asked them to answer the questions based on a one-week recall. However, of the 400 patients enrolled in the study, only 194 provided consent and were available to complete the questionnaire. After excluding patients who did not meet the age criteria of 8-18 years or who completed questionnaires partially, we had a total of 126 patients to include in our analysis (Figure 1). 

Ethical approval

This work received ethical approval from the Institutional Review Board (IRB) of the Jordan University of Science and Technology on 19 February 2023 with the approval number 20230134.

Statistical analysis

In our study, we aimed to compare the HRQoL of celiac disease patients, both males and females, to the normative international average scores provided by the Kidscreen group. We conducted a one-way multivariate analysis of variance (MANOVA) to assess any statistically significant differences between males and females collectively across the 10 health domains.

Then, we divided our sample into four cohorts: celiacs with chronic illnesses versus those without chronic illnesses; celiacs adhering to a strict GFD versus those who are not; celiacs with growth issues versus those without growth issues; and celiacs with a duration of celiac disease lasting more than five years versus those with a duration lasting less than five years. We determined the average T-score and standard deviation for each subgroup. We estimated statistical significance in the form of a p-value at a significance level of less than 0.05, based on the two-sample independent t-test. To provide a more thorough understanding of the findings, we employed a tool to evaluate their practical significance. Because our data were continuous, a modified Cohen's d (Cohen's ds) was used. Additionally, Cohen’s ds for each subgroup was determined along with their 95% confidence intervals. Finally, the Cronbach's alpha value of 0.95 for our sample shows that the Kidscreen-52 questionnaire is very reliable and consistent. It is also close to the upper limit of the questionnaire's range (0.77 to 0.89) that Ravens-Sieberer et al. reported [6].

## Results

Demographics

Our study included 126 patients with an average age of 13.4 years, just over half of whom were females (55.6%). Patients with celiac disease had an average duration of 4.27 years since diagnosis. Regarding the patient strata, roughly two-thirds were strictly adherent to a GFD (61.1%) and had comorbid illnesses (67.5%), while one-third had growth problems (34.1%). Details are available in Table 1.

**Table 1:** Patient demographics

Characteristics	Number (%)
Average Age (Years) ± SD	13.4 ± 2.49
Sex	
Male	56 (44.4)
Female	70 (55.6)
Subgroup analysis	
Average duration since diagnosis (Years) ±SD	4.27 ± 3.09
Patients strictly following a gluten-free diet	77 (61.1)
Have a comorbid illness(es)	85 (67.5)
Have growth problems:	43 (34.1)
Weight < -2SD	9 (7.1)
Height < -2SD	13 (10.3)
Both weight and height < -2SD	21 (16.7)

Quality of life in celiac disease patients

Tables 2-3 contain the average T-scores for both females and males, respectively. The only three measures that showed significantly poorer HRQoL in female patients compared to controls were psychological well-being, social support and peers, and financial resources, with mean T-scores of 37.82, 36.3, and 38.4, respectively. The dimensions of moods and emotions and autonomy stand out because they were very close to statistical significance and only slightly deviated from such a finding with mean T-scores of 38.78 and 39.73, respectively.

**Table 2:** Average T-scores for female patients with celiac disease

Category	International mean (T-score ± SD)	T-score range	Female celiacs (average T-score)	Interpretation
Physical Well-Being	48.5 ± 9.77	38.72-58.27	40.61	Average
Psychological Well-Being	49.36 ± 10.21	39.15-59.57	37.82	Low
Moods & Emotions	48.76 ± 9.99	38.77-58.75	38.78	Average
Self-Perception	47.84 ± 9.87	37.97-57.71	43.26	Average
Autonomy	48.88 ± 10	38.88-58.88	39.73	Average
Parent Relation & Home Life	49.6 ± 10.3	39.3-59.9	46.34	Average
Social Support & Peers	50.37 ± 10.03	40.34-60.4	36.3	Low
School Environment	50.38 ± 9.86	40.52-60.24	45.3	Average
Bullying	50.28 ± 9.95	40.33-60.23	42.42	Average
Financial Resources	49.83 ± 9.98	39.85-59.81	38.40	Low

**Table 3:** Average T-scores for male patients with celiac disease

Category	International mean (T-score for male ± SD)	T-score range	Male celiacs (average T-score)	Interpretation
Physical Well-Being	51.66 ± 10	41.66-61.66	44.85	Average
Psychological Well-Being	50.71 ± 9.71	41-60.42	38.59	Low
Moods & Emotions	51.37 ± 9.83	41.54-61.2	37.57	Low
Self-Perception	52.4 ± 9.58	42.82-61.98	42.81	Low
Autonomy	51.24 ± 9.85	41.39-61.09	41.51	Average
Parent Relation & Home Life	50.44 ± 9.64	40.8-60.08	46.22	Average
Social Support & Peers	49.58 ± 9.95	39.63-59.53	38.86	Low
School Environment	49.57 ± 10.13	39.44-59.7	42.63	Average
Bullying	49.7 ± 10.04	39.66-59.74	39.01	Low
Financial Resources	50.19 ± 10.02	40.17-60.21	38.47	Low

Interestingly, males showed significantly lower HRQoL across a wider range of domains than females did. For example, all variables showed evidence of statistical significance, except for four variables: physical well-being, school environment, parent relation and home life, and autonomy, the latter of which was very close to statistical significance itself. The respective T-scores for these variables were 44.85, 42.63, 46.22, and 41.51.

This was supported by the results of the one-way MANOVA. The p-value for the box test of equality of covariance matrices was less than 0.01, indicating that we cannot reject the null hypothesis that assumes equality of covariance between males and females. Consequently, we utilized a more resilient calculation of MANOVA, notably Pillai's trace. The p-value for Pillai's trace was 0.022, which is below the threshold of 0.05. This discovery strengthens the evidence that there were significant differences in performance between males and females across the 10 areas of HRQoL, with males exhibiting poorer performance.

The influence of concomitant chronic disease on HRQoL

Since 67.5% of our celiac disease patients had concurrent chronic diseases, comparing HRQoL among two strata of patients with and without chronic diseases is valuable. Males with chronic diseases scored significantly lower than those without chronic diseases in two dimensions: moods and emotions (p-value = 0.027) and self-perception (p-value < 0.001). There were no statistically significant differences between the other health dimensions in males and none in females. See Table 4 for a detailed comparison.

**Table 4:** Comparison of quality-of-life scores between celiac disease patients with chronic diseases and those without chronic diseases Two-sample independent t-test, significance level set at p < 0.05.

Category	Females					Males				
	Celiacs with chronic disease - Average T-score ±SD (N=47)	Celiacs with no chronic diseases - Average T-score ±SD (N=23)	P-value	Cohen’s ds (95% CI) for female patients with chronic disease	Cohen’s ds (95% CI) for female patients without chronic disease	Celiacs with chronic disease - Average T-score ±SD (N=19)	Celiacs with no chronic diseases - Average T-score ±SD (N=37)	P-value	Cohen’s ds (95% CI) for male patients with chronic disease	Cohen’s ds (95% CI) for male patients without chronic disease
Physical Well-Being	41.77±8.28	43.63±12.29	0.457	-0.19 (-2.60,2.22)	-0.19 (-5.30,4.92)	41.75±7.69	44.1±10.9	0.41	-0.26 (-2.80,2.27)	-0.26 (-5.28,4.75)
Psychological Well-Being	38.08±7.32	37.53±10.77	0.802	0.06 (-2.07, 2.19)	0.06 (-4.42,4.55)	38.1±7.18	38.66±5.9	0.783	-0.08 (-2.45,2.28)	-0.08 (-2.80,2.63)
Moods & Emotions	37.34±8.61	39.26±13.97	0.451	-0.18 (-2.69, 2.33)	-0.18 (-5.99,5.63)	37.34±1.02	40.03±5.54	0.027	-0.81 (-1.15,-0.48)	-0.81 (-3.36,1.73)
Self-Perception	43.09±8.34	42.22±5.67	0.653	0.11 (-2.31, 2.54)	0.11 (-2.24,2.47)	34.09±8.45	43.16±1.36	<0.001	-1.31 (-4.09,1.48)	-1.31 (-1.93,-0.68)
Autonomy	40.25±6.15	40.18±9.33	0.970	0.01 (-1.78,1.80)	0.01 (-3.87,3.89)	40.25±5.20	40.83±11.56	0.829	-0.07 (-1.79,1.64)	-0.07 (-5.39,5.24)
Parent Relation & Home Life	46.53±9.38	44.78±11.73	0.502	0.17 (-2.56,2.90)	0.17 (-4.71,5.05)	46.25±10.12	45.77±1.16	0.834	0.06 (-3.28,3.39)	0.06 (-0.48,0.59)
Social Support & Peers	37.05±9.69	37.6±8.92	0.82	-0.06 (-2.88,2.76)	-0.06 (-3.77,3.65)	37.11±8.46	38.26±8.46	0.659	-0.14 (-2.92,2.65)	-0.14 (-4.03,3.76)
School Environment	44.38±8.66	42.88±8.64	0.498	0.17 (-2.35,2.69)	0.17 (-3.42,3.77)	44.37±8.51	43.66±4.88	0.744	0.09 (-2.71,2.90)	0.09 (-2.15,2.34)
Bullying	39.62±9.84	43.02±13.47	0.235	-0.31 (-3.17,2.56)	-0.31 (-5.91,5.30)	39.57±9.39	43.7±13.18	0.239	-0.38 (-3.48,2.71)	-0.38 (-6.44,5.68)
Financial Resources	37.3±6.7	38.96±8.24	0.206	-0.23 (-2.18,1.72)	-0.23 (-3.66,3.20)	37.7±6.9	39.65±8.78	0.419	-0.26 (-2.53,2.02)	-0.26 (-4.30,3.78)

Strict GFD and HRQoL

Despite the difficulties in adhering to a strict GFD, 61.1% of our patients indicated proper adherence to therapy. Females who strictly adhered to a GFD demonstrated statistically significant differences exclusively in the parent relation and home life domain (p-value = 0.014). In contrast, no statistically significant differences were observed among males. Refer to Table 5 for a detailed comparison.

**Table 5:** Comparison between quality-of-life between celiac disease patients on a strict GFD vs those not on a strict GFD Two-sample independent t-test, significance level set at p < 0.05. GFD: gluten-free diet

Category	Females					Males				
	Celiacs on strict GFD - Average T-score ±SD (N=44)	Celiacs not following strict GFD - Average T-score ±SD (N=26)	P-value	Cohen’s ds (95% CI) for female patients on a strict GFD	Cohen’s ds (95% CI) for female patients not on a strict GFD	Celiacs on strict GFD - Average T-score ±SD (N=33)	Celiacs not following strict GFD - Average T score ±SD (N=23)	P-value	Cohen’s ds (95% CI) for male patients on a strict GFD	Cohen’s ds (95% CI) for male patients not on a strict GFD
Physical Well-Being	43.12±11.28	41.41±7.21	0.491	0.17 (-3.22,3.56)	0.17 (-2.65,2.99)	43.13±11.36	41.5±7.1	0.545	0.17 (-3.80,4.13)	0.17 (-2.80,3.13)
Psychological Well-Being	39.27±9.31	37.17±7.19	0.327	0.24 (-2.56,3.05)	0.24 (-2.57,3.06)	39.01±8.77	36.82±7.4	0.332	0.27 (-2.79,3.33)	0.27 (-2.83,3.36)
Moods & Emotions	38.66±12.37	37.68±7.85	0.718	0.09 (-3.63,3.81)	0.09 (-2.98,3.16)	38.51±10.7	37.81±7.81	0.790	0.07 (-3.66,3.81)	0.07 (-3.19,3.34)
Self-Perception	43.72±7.18	42.22±8.07	0.423	0.20 (-1.96,2.36)	0.20 (-2.96,3.36)	43.62±7.86	42.18±7.9	0.504	0.18 (-2.56,2.93)	0.18 (-3.12,3.49)
Autonomy	41.64±7.25	40.21±7.52	0.434	0.19(-1.99,2.38)	0.19 (-2.75,3.14)	40.76±6.89	40.13±7.38	0.745	0.09 (-2.32,2.49)	0.09 (-3.00,3.17)
Parent Relation & Home Life	48.86±9.64	43.72±10.06	0.014	0.52 (-2.38,3.42)	0.52 (-3.41,4.46)	48.09±11.36	43.44±9.94	0.119	0.43 (-3.53,4.40)	0.43 (-3.72,4.59)
Social Support & Peers	38.42±8.26	36.40±11.03	0.387	0.22 (-2.27,2.70)	0.22 (-4.10,4.53)	38.26±7.35	36.48±10.81	0.466	0.20 (-2.37,2.76)	0.20 (-4.32,4.72)
School Environment	44.79±9.13	43.5±7.8	0.549	0.15(-2.60,2.90)	0.15 (-2.90,3.20)	44.53±8.85	43.51±7.46	0.653	0.12 (-2.97,3.21)	0.12 (-3.00,3.24)
Bullying	41.5±11.52	40.64±11.06	0.760	0.08 (-3.39,3.54)	0.08 (-4.25,4.40)	41.14±11.25	40.55±10.84	0.845	0.05 (-3.87,3.98)	0.05 (-4.48,4.58)
Financial Resources	39.67±7.4	36.87±6.91	0.122	0.39 (-1.84,2.61)	0.39 (-2.32,3.09)	37.7±6.9	39.65±8.78	0.187	0.36 (-2.28,3.00)	0.36 (-2.52,3.25)

Growth problems and HRQoL

A total of 34.1% of our patients had growth problems, defined as a weight 2 SD below the mean, a height 2 SD below the mean, or both. Two dimensions, school environment (p-value = 0.029) and financial resources (p-value = 0.045), demonstrated statistically significant differences across health domains in females. No other domain exhibited statistically significant differences in females, and none exhibited statistically significant differences in males. See Table 6 for a detailed comparison.

**Table 6:** Comparison of quality-of-life scores between celiac disease patients with growth issues versus those without growth issues Two-sample independent t-test, significance level set at p < 0.05.

Category	Females					Males				
	Celiacs with growth issues - Average T-score ±SD (N=22)	Celiacs with no growth issues - Average T-score ±SD (N=48)	P-value	Cohen’s ds (95% CI) for female patients on with growth issues	Cohen’s ds (95% CI) for female patients on without growth issues	Celiacs with growth issues - Average T-score ±SD (N=21)	Celiacs with no growth issues - Average T-score ±SD (N=35)	P-value	Cohen’s ds (95% CI) for male patients on with growth issues	Cohen’s ds (95% CI) for male patients on without growth issues
Physical Well-Being	44.08±9.58	39.43±9.56	0.064	-0.49 (-4.55,3.58)	-0.49 (-3.24,2.27)	44.12±9.63	39.63±9.41	0.089	-0.47 (-4.59,3.65)	-0.47 (-3.73,2.79)
Psychological Well-Being	39.31±8.25	36.34±8.79	0.176	-0.35 (-4.09,3.39)	-0.35 (-2.73,2.02)	39.04±8.15	36.01±8.93	0.192	-0.36 (-4.27,3.55)	-0.36 (-3.12,2.40)
Moods & Emotions	39.98±9.93	34.76±11.33	0.056	-0.50 (-5.32,4.32)	-0.50 (-3.36,2.36)	39.62±9.38	34.83±11.07	0.084	-0.48 (-5.32,4.37)	-0.48 (-3.66,2.70)
Self-Perception	43.45±7.89	42.39±6.63	0.656	-0.14 (-2.96,2.68)	-0.14 (-2.41,2.13)	43.46±7.93	42.37±6.48	0.593	-0.15 (-2.98,2.69)	-0.15 (-2.83,2.54)
Autonomy	41.18±6.62	39.34±8.51	0.332	-0.25 (-3.87,3.37)	-0.25 (-2.16,1.65)	41.09±6.67	39.36±8.31	0.386	-0.24 (-3.87,3.40)	-0.24 (-2.50,2.02)
Parent Relation & Home Life	47.58±11.05	44.25±8.72	0.214	-0.32 (-4.03,3.39)	-0.32 (-3.50,2.86)	47.22±10.99	44.11±8.59	0.247	-0.31 (-4.06,3.45)	-0.31 (-4.03,3.42)
Social Support & Peers	38.31±9.55	35.99±8.96	0.275	-0.25 (-4.06,3.56)	-0.25 (-3.00,2.50)	38.33±9.59	36.21±8.82	0.407	-0.23 (-4.09,3.63)	-0.23 (-3.48,3.02)
School Environment	45.77±8.85	41.01±7.13	0.029	-0.57 (-3.60,2.46)	-0.57 (-3.12,1.98)	45.54±8.8	41.17±7.00	0.054	-0.53 (-3.60,2.53)	-0.53 (-3.52,2.45)
Bullying	41.88±11.14	40.06±11.6	0.534	-0.16 (-5.10,4.77)	-0.16 (-3.37,3.05)	41.14±11.02	39.91±11.35	0.686	-0.11 (-5.08,4.86)	-0.11 (-3.84,3.62)
Financial Resources	39.76±7.06	36.04±7.06	0.045	-0.53 (-3.53,2.48)	-0.53 (-2.56,1.51)	39.48±7.14	36.2±6.92	0.134	-0.46 (-3.49,2.56)	-0.46 (-2.88,1.96)

The average duration since diagnosis and HRQoL

Notably, our sample had an average duration of 4.27 years since celiac disease diagnosis. Accordingly, we stratified patients into those with celiac disease for less than five years and those with celiac disease for more than five years. For both males and females, the 10 dimensions of HRQoL showed no statistically significant differences between the two strata. See Table 7 for a detailed comparison.

**Table 7:** Comparison of quality-of-life scores between celiac disease patients based on average years of diagnosis with a five-year cut off Two-sample independent t-test, significance level set at p < 0.05.

Category	Females					Males				
	Celiacs for more than 5 years - Average T-score ±SD (N=32)	Celiacs for less than 5 years - Average T-score ±SD (N=38)	P-value	Cohen’s ds (95% CI) for female patients with celiac disease for more than 5 years	Cohen’s ds (95% CI) for female patients with celiac disease for less than 5 years.	Celiacs for more than 5 years - Average T-score ±SD (N=22)	Celiacs for less than 5 years - Average T-score ±SD (N=34)	P-value	Cohen’s ds (95% CI) for male patients with celiac disease > 5 years	Cohen’s ds (95% CI) for male patients with celiac disease < 5 years
Physical Well-Being	44.24±9.76	41.12±9.79	0.188	0.32 (-3.12,3.76)	0.32 (-2.85,3.49)	44.17±9.68	41.24±9.56	0.270	0.29 (-3.85,4.42)	0.29 (-3.00,3.57)
Psychological Well-Being	39.11±9.30	37.89±7.93	0.556	0.14 (-3.14,3.42)	0.14 (-2.42,2.71)	39.07±9.22	37.47±8.09	0.497	0.18 (-3.76,4.12)	0.18 (-2.60,2.96)
Moods & Emotions	38.13±10.83	38.38±10.77	0.923	-0.02 (-3.84,3.80)	-0.02 (-3.51,3.46)	38.3±10.8	38.19±10.5	0.970	0.01 (-4.61,4.63)	0.01 (-3.60,3.62)
Self-Perception	44.88±7.07	41.81±7.63	0.088	0.42 (-2.08,2.91)	0.42 (-2.05,2.89)	44.83±7.01	41.74±7.43	0.126	0.40 (-2.60,3.39)	0.40 (-2.16,2.95)
Autonomy	41.88±6.8	39.76±7.61	0.227	0.29 (-2.11,2.69)	0.29 (-2.17,2.76)	41.8±6.77	39.56±7.51	0.263	0.29 (-2.60,3,19)	0.29 (-2.29,2.88)
Parent Relation & Home Life	48.54±9.52	45.32±10.42	0.185	0.32 (-3.04,3.68)	0.32 (-3.05,3.69)	48.32±9.56	44.76±10.71	0.211	0.31 (-3.77,4.40)	0.31 (-3.37,4.00)
Social Support & Peers	39.27±9.95	36.36±8.86	0.200	0.31 (-3.20,3.82)	0.31 (-2.56,3.18)	39.22±9.86	36.34±8.69	0.256	0.31 (-3.91,4.52)	0.31 (-2.68,3.30)
School Environment	45.7±9.93	43.09±7.28	0.210	0.30 (-3.20,3.81)	0.30 (-2.05,2.66)	45.64±9.84	43.01±7.12	0.252	0.30 (-3.91,4.51)	0.30 (-2.15,2.75)
Bullying	41.54±11.71	40.79±10.99	0.81	0.07 (-4.06,4.20)	0.07 (-3.49,3.62)	41.48±11.61	40.48±10.86	0.745	0.08 (-4.88,5.05)	0.08 (-3.65,3.82)
Financial Resources	39.65±7.76	37.65±6.89	0.258	0.27 (-2.46,3.01)	0.27 (-1.96,2.50)	39.45±7.78	37.67±6.7	0.366	0.24 (-3.08,3.50)	0.24 (-2.06,2.55)

## Discussion

It is worth noting that several authors have previously addressed the quality of life of celiac disease patients, without reaching a true consensus on the actual impact of celiac disease [13,14]. The heterogeneous nature of those studies, including but not limited to different inclusion criteria, the use of self vs. proxy assessments, and the various types of questionnaires used by these studies, could account for this.

Our findings shed light on the decrease in HRQoL among children with celiac disease. Haj Ali et al. in Jordan published a paper indicating that adults with celiac disease are more likely to experience anxiety and depressive symptoms compared to the general population [15]. These authors did not conduct a thorough HRQoL evaluation of these patients, but their results are consistent with ours in that patients with celiac disease, whether male or female, bear a significant psychological burden. Although Haj Ali and colleagues conducted their study on adults with celiac disease, our work stands out as the first in Jordan to assess HRQoL among children with celiac disease.

Males reported a significantly lower HRQoL than females, which is in contrast to an Italian study by Altobelli and colleagues that found an equal distribution of disease burden between the two sexes [14]. Reports from the Western world also contradict our findings in the sense that females in their respective populations demonstrate lower HRQoL scores [8]. Interestingly, our data are in line with a study from Saudi Arabia reporting a lower HRQoL among the male population [16]. Cultural differences between the Arab world and the Western community could account for this, as females in the Arab world participate in fewer recreational social activities than their male counterparts. A study by Allison and colleagues supports this. When stratified according to sex, Jordanian men had a greater number of recreation groups than their female counterparts [17]. This means that males are more likely to deal with issues related to self-perception and daily physical comparisons. Our findings provide more evidence in favor of this theory, as only males displayed a lower HRQoL in the bullying and self-perception domains.

Regarding the influence of a strict GFD on HRQoL, we only found that females had significantly lower scores across one domain, namely parent relation and home life, whereas males did not. Wagner et al.'s paper, which demonstrated a significantly lower quality of life among patients without proper dietary adherence, contradicts this finding [18]. One possible explanation for this discrepancy is the ambiguity surrounding the definition of dietary adherence. Gluten is found in a myriad of food products. This, combined with the lack of proper food labeling and community-based awareness about gluten-free products, could mean that some children might unknowingly consume gluten despite believing they are adherent [19].

Using a five-year cutoff point, our study also revealed no difference in HRQoL according to the average number of years since diagnosis. A Brazilian study that assessed the quality of life among an adult celiac population supports this [20]. Importantly, Castilhos and colleagues observed that the average number of years since diagnosis implies a GFD for most of their population, which could explain the lack of statistical significance in this regard. This could also be the case in our report, given that approximately two-thirds of our population is already adhering to a GFD, reducing the impact of the disease's duration.

Interestingly, our results showed that males with chronic diseases performed worse in terms of moods and emotions and self-perception than males without other chronic illnesses. A paper by Carta et al. studied the impact of affective disorders, particularly major depressive disorder, panic disorder, and bipolar disorder, on the HRQoL of celiac disease patients. Their findings showed that these comorbidities play a critical role in impairing the daily lives of patients with celiac disease [21]. Notably, the only areas exhibiting lower HRQoL are affective, notably moods and emotions as well as self-perception, although affective diseases are not among the chronic illnesses addressed in our study.

Despite its several strengths, including being the first paper to investigate the quality of life among Jordanian children with celiac disease and its potential influence on GFD legislation, our study was subject to a few limitations. First, the Kidscreen group provided normative data for the 13 European countries in which the questionnaire was concurrently designed, none of which was provided for Jordan. Second, as suggested by Barrio and colleagues, it could be better to use both a generic and a disease-specific questionnaire in addressing the quality of life among celiac disease patients, as explained by the greater sensitivity of the disease-specific questionnaires [22]. In our study, we only used the generic Kidscreen-52 questionnaire. Egilson et al. assessed the quality of life among patients with autism spectrum disorder using a shortened version of the Kidscreen-52 questionnaire, namely, the 27th version. The author suggested using both the self-evaluation and parent proxy assessment versions of the questionnaire to provide a more comprehensive assessment of the children's quality of life [23]. We employed only the self-assessment version. Lastly, the fact that all our patients belonged to a single celiac disease society could potentially introduce bias, as they may not accurately represent patients not enrolled in a celiac disease society, who may experience a less severe impact from the disease or lack proper logistical access to such societies.

## Conclusions

To the best of our knowledge, no study has previously addressed the impact of growth issues on the quality of life of celiac disease patients. Compared to the general population, individuals with celiac disease frequently have lower scores for height, weight, or both. Our study revealed that one-third of the patients reported this issue. This can be attributed to celiac disease being a multisystem disorder, with potential endocrine sequelae stemming from its intrinsic pathophysiology and the nutritional challenges imposed by a GFD. Our study also revealed poorer HRQoL among females with celiac disease and growth problems in two domains, namely, school environment and financial resources. We can attribute the latter to the high cost of GFD. Regretfully, there is still a severe dearth of appropriate laws that would allow patients to receive GFDs, and even in such cases, the importance of having a qualified nutritionist cannot be overstated.

In light of these findings, several policies can be implemented to improve the HRQoL of children with celiac disease. Firstly, enforcing clear food labeling and offering gluten-free meal options in schools are both valuable initiatives, as they greatly assist patients and their parents in adhering to a strict GFD. Given the high cost of GFDs, expanding insurance coverage to include gluten-free foods would make this diet more affordable and accessible for families. Furthermore, since celiac patients often face significant psychosocial challenges, providing teacher training is vital for fostering a more inclusive school environment. Public awareness campaigns are also crucial to creating a society that is more understanding of individuals with celiac disease. Future research should focus on evaluating the efficacy of the proposed policy changes in improving dietary adherence, accessibility, and psychosocial well-being among children with celiac disease. This would provide evidence-based insights to guide future policy changes.
